# Supplementary material for: Cost-effectiveness analysis of alternative infant and neonatal rotavirus vaccination schedules in Malawi
Source: PLOS Glob Public Health. 2025 Apr 10;5(4):e0004341. doi: 10.1371/journal.pgph.0004341 (PMC11984971; doi:10.1371/journal.pgph.0004341)
Supplement: S1 Fig — (DOCX) [file pgph.0004341.s002.docx]

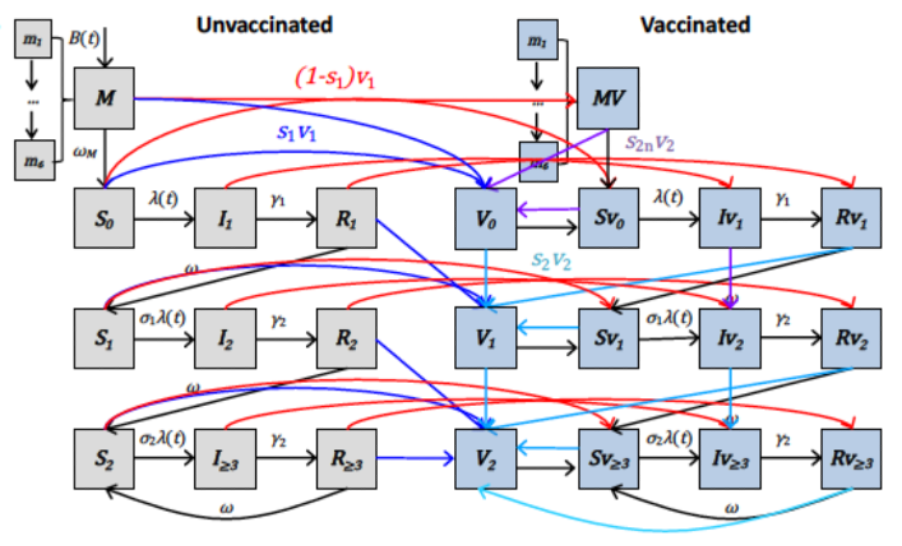


**S1 Fig. Diagram of the transmission dynamic model.** Compartments in grey and blue represent unvaccinated and vaccinated individuals, respectively. The arrows represent individuals transitioning between states. Blue arrows represent individuals who respond to the first (dark blue) and subsequent (line blue) vaccine doses, while red arrows represent individuals who fail to respond to the first vaccine dose; purple arrows represent individuals who did not respond to the first dose, but did respond to a subsequent dose (assuming heterogeneity in vaccine response).
