# Supplementary material for: Cost-effectiveness analysis of alternative infant and neonatal rotavirus vaccination schedules in Malawi
Source: PLOS Glob Public Health. 2025 Apr 10;5(4):e0004341. doi: 10.1371/journal.pgph.0004341 (PMC11984971; doi:10.1371/journal.pgph.0004341)
Supplement: S2 Fig — (DOCX) [file pgph.0004341.s003.docx]

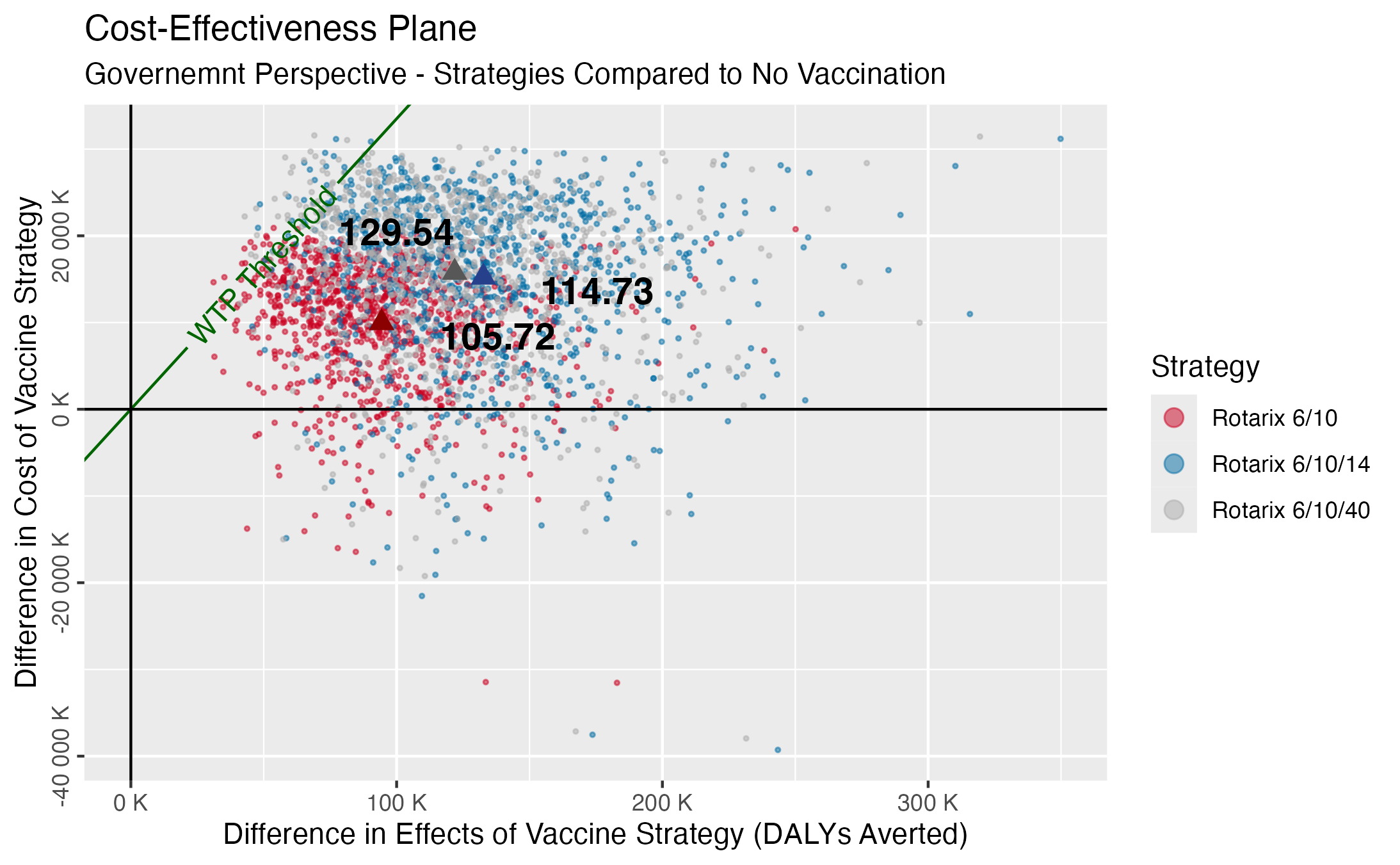


**S2 Fig.** **Cost-effectiveness plane for the Rotarix strategies compared to no vaccination from the government perspective.** Individual circular points represent each simulation’s incremental costs (y-axis) and incremental effects (in terms of disability-adjusted life-years (DALYs) averted, x-axis) compared to no vaccination. The triangular points represent the average cost-effectiveness ratio (incremental costs over incremental effects) for each strategy compared to no vaccination. The green line represents a willingness-to-pay (WTP) threshold of $335 per DALY averted (0.5x Malawi’s GDP per capita); points to the right of and below the WTP threshold are considered cost-effective compared to no vaccination.
