# Supplementary material for: Cost-effectiveness analysis of alternative infant and neonatal rotavirus vaccination schedules in Malawi
Source: PLOS Glob Public Health. 2025 Apr 10;5(4):e0004341. doi: 10.1371/journal.pgph.0004341 (PMC11984971; doi:10.1371/journal.pgph.0004341)
Supplement: S4 Fig — (DOCX) [file pgph.0004341.s005.docx]

**
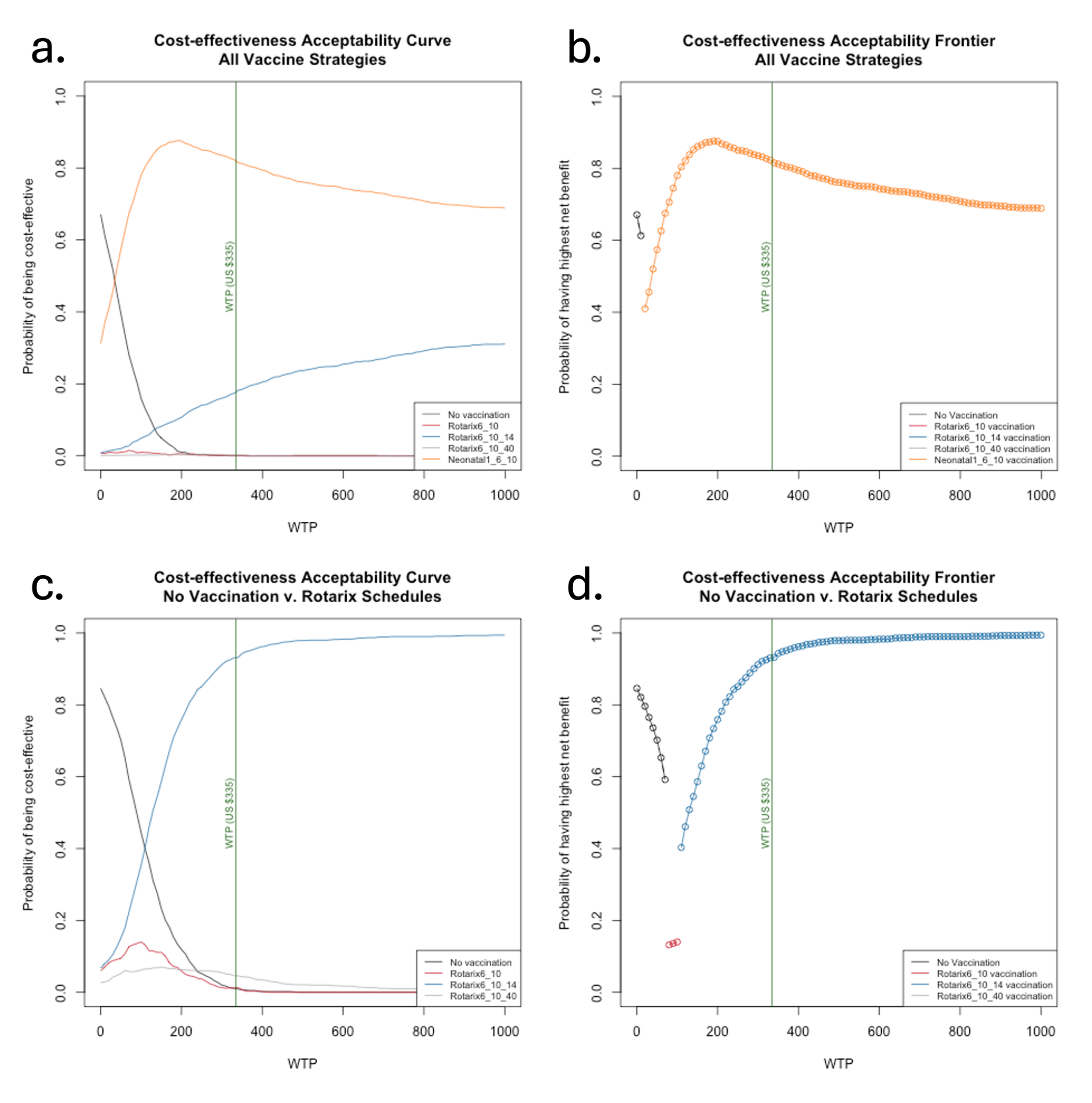
S4 Fig. Cost-effectiveness acceptability curves and frontier for all strategies and for strategies currently available on the market from the societal perspective.** The cost-effectiveness acceptability curves for (a) all strategies and (c) the currently available strategies show the probability that each strategy will produce the highest net benefit compared to all other strategies (y-axis) for a range of willingness-to-pay (WTP) values (x-axis), whereas the cost-effectiveness acceptability frontier for (b) all strategies and (d) the currently available strategies show the probability that the strategy with the highest average net benefit is preferred, highlighting the level of certainty in the optimal strategy based on how much decision-makers are willing to pay for one disability-adjusted life-year averted. The vertical green line marks the WTP threshold set at 0.5x Malawi’s gross domestic product per capita.
