# Supplementary material for: Cost-effectiveness analysis of alternative infant and neonatal rotavirus vaccination schedules in Malawi
Source: PLOS Glob Public Health. 2025 Apr 10;5(4):e0004341. doi: 10.1371/journal.pgph.0004341 (PMC11984971; doi:10.1371/journal.pgph.0004341)
Supplement: S6 Fig — (DOCX) [file pgph.0004341.s007.docx]

**
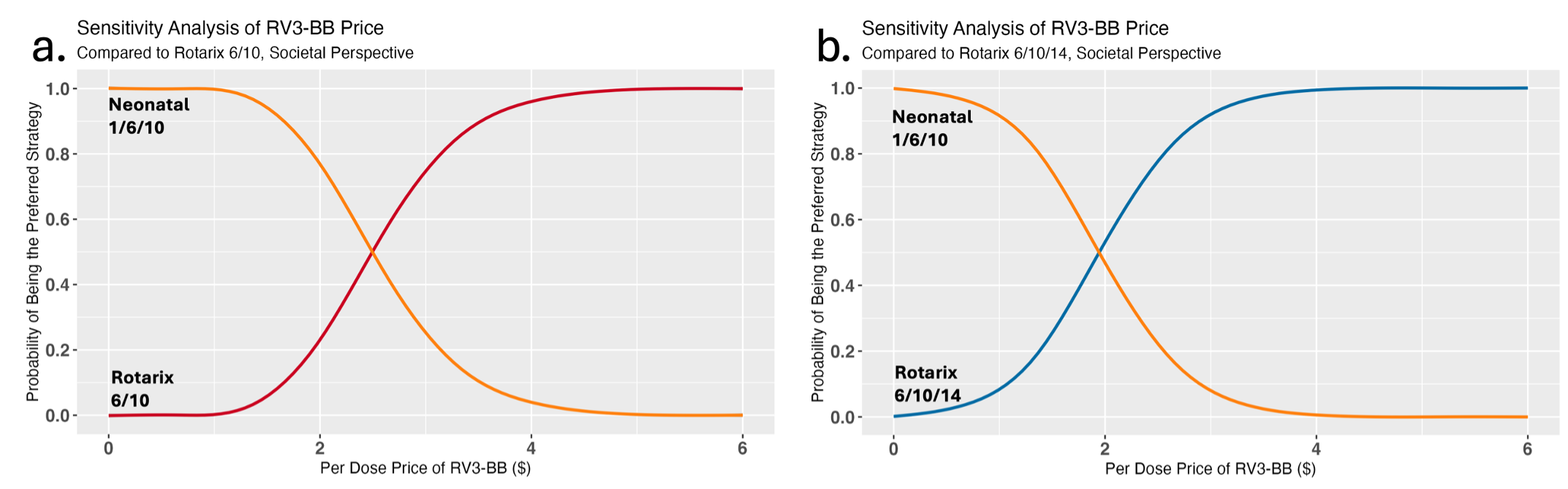
S6 Fig. Sensitivity analysis of the price per dose of the RV3-BB neonatal vaccine from the societal perspective.** The probability that the neonatal vaccine will be cost-effective (i.e. preferred) compared to (a) the current Rotarix schedule and (b) the optimal three-dose Rotarix schedule is plotted on the y-axis depending on the price per dose (on the x-axis). The RV3-BB neonatal schedule is depicted in orange, the current Rotarix 6/10 schedule in red, and the Rotarix 6/10/14 schedule in blue. The Rotarix price remained fixed at $1.94 per dose. The willingness-to-pay (WTP) threshold is set at $335 per DALY averted.
