# Supplementary material for: Cost-effectiveness analysis of alternative infant and neonatal rotavirus vaccination schedules in Malawi
Source: PLOS Glob Public Health. 2025 Apr 10;5(4):e0004341. doi: 10.1371/journal.pgph.0004341 (PMC11984971; doi:10.1371/journal.pgph.0004341)
Supplement: S1 Table — (DOCX) [file pgph.0004341.s008.docx]

**S1 Table. The fixed, fitted, and estimated parameter definitions and their sources for the dynamic model.** The values in the brackets for the fitted parameters represent the 95% credible intervals. The values in parentheses for the estimated parameters (proportion of individuals who respond to each dose of the vaccine) represent ±20% around the mean value.

| **Parameter** | **Symbol** | **Value** | **source** |
| --- | --- | --- | --- |
| ***Fixed parameters*** |  |  |  |
| Average duration of maternal immunity | 1/*ω*_m_ | 26 weeks | [1,2] |
| Duration of primary infection | 1/*γ1* | 1 week | [3] |
| Duration of subsequent infection | 1/*γ*_2_ | 0.5 week | [4,5] |
| Relative risk of second infection | *σ*_1_ | 0.62 | [6,7] |
| Relative risk of third infection | *σ*_2_ | 0.35 | [6,7] |
| Relative infectiousness of secondary infection | *ρ*_2_ | 0.5 | [6,7] |
| Relative infectiousness of mild/asymptomatic infections | *ρ*_≥3_ | 0.1 | [8] |
| ***Fitted parameters*** |  |  |  |
| Basic reproductive number | *R*_0_ | 78.8 (70.5-96.2) | [9] |
| Amplitude of seasonal forcing | *b* | 0.174 (0.113-0.294) | [9] |
| Seasonal offset (weeks) | *φ* | 6.9 (4.0-11.2) | [9] |
| Proportion of moderate-to-severe diarrhea cases reported | *h* | 0.017 (0.016-0.018) | [9] |
| Duration of vaccine-induced immunity (weeks) | *ω_ν_* | 45.260 (32.172-85.514) | [9] |
| ***Estimated parameters*** |  |  |  |
| **Rotarix vaccine** |  |  |  |
| Proportion who responded to the first dose | *S_C1_* | 0.527 (0.422-0.632) | [10] |
| Proportion who responded to the second dose | *S_C2_* | 0.895 (0.716-1.00) | [10] |
| Proportion who responded to the third dose | *S_C3_* | 0.895 (0.716-1.00) | [10] |
| Proportion who responded to the second dose given that they failed to respond to the first dose | *S_C2n_* | 0.117 (0.094-0.140) | [10] |
| Proportion who responded to the third dose given that they failed to respond to either first or second dose | *S_C3n_* | 0.014 (0.011-0.017) | [10] |
| **RV3-BB vaccine** |  |  |  |
| Proportion who responded to the first dose | *S_C1_* | 0.608 (0.486-0.730) | [11] |
| Proportion who responded to the second dose | *S_C2_* | 0.641 (0.513-0.769) | [11] |
| Proportion who responded to the third dose | *S_C3_* | 0.641 (0.513-0.769) | [11] |
| Proportion who responded to the second dose given that they failed to respond to the first dose | *S_C2n_* | 0.558 (0.446-0.670) | [11] |
| Proportion who responded to the third dose given that they failed to respond to either first or second dose | *S_C3n_* | 0.453 (0.362-0.544) | [11] |

# References

1. Pitzer VE, Viboud Cc, Lopman BA, Patel MM, Parashar UD, Grenfell BT. Influence of birth rates and transmission rates on the global seasonality of rotavirus incidence. Journal of the Royal Society Interface. 2011;8:10.

2. Patel M, Shane AL, Parashar UD, Jiang B, Gentsch JR, Glass RI. Oral rotavirus vaccines: how well will they work where they are needed most? J Infect Dis. 2009;200 Suppl 1(0 1):S39-48.

3. Kambhampati A, Payne DC, Costantini V, Lopman BA. Host Genetic Susceptibility to Enteric Viruses: A Systematic Review and Metaanalysis. Clinical Infectious Diseases. 2016;62(1):8.

4. Ultsch B, Damm O, Beutels P, Bilcke J, Bru¨ggenju¨rgen B, Gerber-Grote A, et al. Methods for Health Economic Evaluation of Vaccines and Immunization Decision Frameworks: A Consensus Framework from a European Vaccine Economics Community. PharmacoEconomics. 2015;34:17.

5. Gelman A, Rubin DB. Inference from Iterative Simulation Using Multiple Sequences. Statistical Science. 1992;7(4):16.

6. Velázquez FR, Matson DO, Calva JJ, Guerrero L, Morrow AL, Carter-Campbell S, et al. Rotavirus infection in infants as protection against subsequent infections. New England Journal of Medicine. 1996;335(14):7.

7. Gladstone BP, Ramani S, Mukhopadhya I, Muliyi J, Sarkar R, Rehman AM, et al. Protective effect of natural rotavirus infection in an Indian birth cohort. New England Journal of Medicine. 2011;365(4):10.

8. Van Effelterre T, Soriano-Gabarró M, Debrus S, Newbern EC, Gray J. A mathematical model of the indirect effects of rotavirus vaccination. Epidemiology & Infection. 2010;138(6):14.

9. Pitzer VE, Bennett A, Bar-Zeev N, Jere KC, Lopman BA, Lewnard JA, et al. Evaluating strategies to improve rotavirus vaccine impact during the second year of life in Malawi. Science Translational Medicine. 2019;11:12.

10. Cunliffe NA, Witte D, Ngwira BM, Todd S, Bostock NJ, Turner AM, et al. Efficacy of human rotavirus vaccine against severe gastroenteritis in Malawian children in the first two years of life: a randomised, double-blind, placebo controlled trial. Vaccine. 2012;30(1):16.

11. Witte D, Handley A, Jere KC, Bogandovic-Sakran N, Mpakiza A, Turner A, et al. Neonatal rotavirus vaccine (RV3-BB) immunogenicity and safety in a neonatal and infant administration schedule in Malawi: a randomised, double-blind, four-arm parallel group dose-ranging study. Lancet Infectious Disease. 2022;22:11.
