# Supplementary material for: Cost-effectiveness analysis of alternative infant and neonatal rotavirus vaccination schedules in Malawi
Source: PLOS Glob Public Health. 2025 Apr 10;5(4):e0004341. doi: 10.1371/journal.pgph.0004341 (PMC11984971; doi:10.1371/journal.pgph.0004341)
Supplement: S2 Table — (DOCX) [file pgph.0004341.s009.docx]

**S2 Table. Total number of cases, hospitalizations, DALYs, and deaths that occurred during the 2025-2035 period for each vaccine simulation.**

| Outcomes by Vaccine Schedule | | | | | | |
| --- | --- | --- | --- | --- | --- | --- |
| Total Outcomes per Strategy | | | | | | |
| Strategy | Total Cases (millions) | Non-Severe Cases (millions) | Moderate-to-Severe Cases (thousands) | Hospitalizations (thousands) | DALYs* (thousands) | Deaths |
| No vaccine | 12.4 (11.3,13.9) | 11.5 (10.4,13.0) | 905.5 (901.9,909.1) | 434.6 (418.8,451.5) | 358.1 (186.9,580.7) | 10600 (5210,17648) |
| Neonatal 1/6/10 | 9.8 (7.9,11.9) | 9.3 (7.4,11.4) | 542.2 (450.4,618.2) | 260.2 (217.8,299.9) | 223.0 (119.4,362.4) | 6400 (3125,10688) |
| Rotarix 6/10 | 10.9 (9.1,12.6) | 10.2 (8.5,11.9) | 650.9 (581.1,720.5) | 312.4 (276.8,348.8) | 263.7 (143.6,424.2) | 7600 (3688,12598) |
| Rotarix 6/10/14 | 9.9 (8.2,11.8) | 9.4 (7.7,11.2) | 548.9 (472.9,630.3) | 263.5 (226.0,303.1) | 225.4 (124.5,362.4) | 6400 (3081,10703) |
| Rotarix 6/10/40 | 9.9 (8.0,11.8) | 9.3 (7.5,11.2) | 580.4 (494.8,660.9) | 278.6 (237.0,319.3) | 236.2 (129.0,377.3) | 6800 (3258,11254) |
| All outcome means are presented with 95% Prediction Intervals | | | | | | |
| *DALYs are discounted at a rate of 3% per year | | | | | | |
