# Supplementary material for: Cost-effectiveness analysis of alternative infant and neonatal rotavirus vaccination schedules in Malawi
Source: PLOS Glob Public Health. 2025 Apr 10;5(4):e0004341. doi: 10.1371/journal.pgph.0004341 (PMC11984971; doi:10.1371/journal.pgph.0004341)
Supplement: S3 Table — (DOCX) [file pgph.0004341.s010.docx]

**S3 Table. Number of cases, hospitalizations, DALYs, and deaths averted by each strategy during the 2025-2035 period for each vaccine simulation compared to the current Rotarix 6/10 schedule.**

| Outcomes Averted by Vaccine Schedule | | | | | | |  |
| --- | --- | --- | --- | --- | --- | --- | --- |
| Averted Outcomes per Strategy Compared to Rotarix 6/10 | | | | | | |  |
| Strategy | Cases Averted (millions) | Non-Severe Cases Averted (millions) | Moderate-to-Severe Cases Averted (thousands) | Hospitalizations Averted (thousands) | DALYs* Averted (thousands) | Deaths Averted | |
| No vaccine | -1.5 | -1.3 | -254.6 | -122.2 | -94.4 | -3000 | |
| Neonatal 1/6/10 | 1.1 | 0.9 | 108.7 | 52.2 | 40.7 | 1200 | |
| Rotarix 6/10 | -- | -- | -- | -- | -- | -- | |
| Rotarix 6/10/14 | 1.0 | 0.8 | 102.0 | 48.9 | 38.3 | 1200 | |
| Rotarix 6/10/40 | 1.0 | 0.9 | 70.5 | 33.8 | 27.5 | 800 | |
| **DALYs are discounted at a rate of 3% per year* | | | | | | |  |
