# Supplementary material for: Cost-effectiveness analysis of alternative infant and neonatal rotavirus vaccination schedules in Malawi
Source: PLOS Glob Public Health. 2025 Apr 10;5(4):e0004341. doi: 10.1371/journal.pgph.0004341 (PMC11984971; doi:10.1371/journal.pgph.0004341)
Supplement: S4 Table — (DOCX) [file pgph.0004341.s011.docx]

**S4 Table. Summary of the dosing schedules, number of doses, and age of vaccination.**

| **Schedule (weeks)** | **Number of doses** | **Age at vaccination (months)** |
| --- | --- | --- |
| 6/10 | 2 | 2, 3 |
| 6/10/14 | 3 | 2, 3, 4 |
| 6/10/40 | 3 | 2, 3, 9 |
| 1/6/10 | 3 | 0, 2, 3 |
