# Supplementary material for: Cost-effectiveness analysis of alternative infant and neonatal rotavirus vaccination schedules in Malawi
Source: PLOS Glob Public Health. 2025 Apr 10;5(4):e0004341. doi: 10.1371/journal.pgph.0004341 (PMC11984971; doi:10.1371/journal.pgph.0004341)
Supplement: S5 Table — (DOCX) [file pgph.0004341.s012.docx]

**S5 Table. DALYs averted and incremental cost-effectiveness ratios for all vaccine strategies compared to the current Rotarix 6/10 schedule from the societal perspective.**

| Cost-Effectiveness Comparison of All Vaccine Strategies | | | | | |
| --- | --- | --- | --- | --- | --- |
| Societal perspective | | | | | |
| Strategy | Cost (millions) | DALYs (thousands) | Incremental Cost (millions) | DALYs Averted (thousands) | ICER ($/DALY averted) vs next best alternative |
| No vaccine | $101.1 | 358.1 | --- | --- | --- |
| Neonatal 1/6/10 | $103.7 | 223.0 | $2.6 | 135.1 | $19 |
| Rotarix 6/10 | $108.5 | 263.7 | $4.8 | -40.70 | **Dominated** |
| Rotarix 6/10/14 | $112.6 | 225.4 | $8.9 | -2.40 | **Dominated** |
| Rotarix 6/10/40 | $113.4 | 236.2 | $9.7 | -13.20 | **Dominated** |
| Costs reflect 2025 USD | | | | | |
| In conformity with accepted practice, all incremental costs and DALYs averted are computed compared to the next smallest, non-dominated strategy | | | | | |
