# Supplementary material for: Cost-effectiveness analysis of alternative infant and neonatal rotavirus vaccination schedules in Malawi
Source: PLOS Glob Public Health. 2025 Apr 10;5(4):e0004341. doi: 10.1371/journal.pgph.0004341 (PMC11984971; doi:10.1371/journal.pgph.0004341)
Supplement: S6 Table — (DOCX) [file pgph.0004341.s013.docx]

**S6 Table. DALYs averted and incremental cost-effectiveness ratios for available vaccine strategies compared to no vaccination from the societal perspective.**

| Cost-Effectiveness Comparison of Available Vaccine Strategies | | | | | |
| --- | --- | --- | --- | --- | --- |
| Societal perspective | | | | | |
| Strategy | Cost (millions) | DALYs (thousands) | Incremental Cost (millions) | DALYs Averted (thousands) | ICER ($/DALY averted) vs next best alternative |
| No vaccine | $101.1 | 358.1 | --- | --- | --- |
| Rotarix 6/10 | $108.5 | 263.7 | $7.4 | 94.4 | $78 |
| Rotarix 6/10/14 | $112.6 | 225.4 | $4.1 | 38.3 | $107 |
| Rotarix 6/10/40 | $113.4 | 236.2 | $0.8 | -10.8 | **Dominated** |
| Costs reflect 2025 USD | | | | | |
| In conformity with accepted practice, all incremental costs and DALYs averted are computed compared to the next smallest, non-dominated strategy | | | | | |
