# Supplementary material for: Cost-effectiveness analysis of alternative infant and neonatal rotavirus vaccination schedules in Malawi
Source: PLOS Glob Public Health. 2025 Apr 10;5(4):e0004341. doi: 10.1371/journal.pgph.0004341 (PMC11984971; doi:10.1371/journal.pgph.0004341)
Supplement: Attachment — Submitted filename: Notes.docx [file pgph.0004341.s015.docx]

Thank you for allowing me to review this work. I enjoyed the opportunity to learn a lot from the modeling the team performed. While I am not trained in modeling to this level, I did read this from a perspective as a practicing ID specialist with time overseas supporting community epi and economic work to support potential vaccine study. I think the paper is great, and hope that my input is helpful. The below suggestions are largely simple suggestions to consider. Great work!

Line 28-This aim statement is a little confusing, but becomes clear in the introduction, consider using a version of the background primary aim statement.

Line 39- Attempt better parallel description of the findings. I’m a little confused by the results and the way they are summarized leading up to the final estimated cost/DALY for the neonatal vaccine. Would it be permissible to match the cost/DALY for the respective current Rotarix regimen and the studied phase II regimen, and then transition to a statement comparing cost differences in savings to achieve similar benefit on the population/societal level? It reads as though the new vaccine would result in a lower benefit than keeping with and adding in a booster dose.

Line 83- References a slightly different primary aim than that mentioned in the abstract, consider replacing line 28 with this aim statement inserting the Rotarix 2 dosing strategy comparison

Line 86- Almost reads like it would be better fit in the methods section, discussion of how you down selected regimens to study at this point seems premature. I think it may read better if the reasons you chose this aim are listed without mention of this process in this section.

Line 239 consider qualifying this 6/10/40 statement with “additional” or similar adjectives to help

Line 242- Are these 1.1 million additional cases averted? Apologies, I’m a clinician and trying to move this quickly, qualifying the values as additional or less, helps me put it into context of the concerns raised about a plateauing of impact. The way it reads now, I may be trading averted cases for cost savings. Visually in the violin plots it looks like it maybe outperforms the other 3 dose regimens.

Methods- It may be helpful to define threshold for “dominate” strategy like establishing thresholds for statistical significance. Again, this comes from the standpoint of a clinically active ID specialist, and this may be common vernacular for conformity modeling. Feel free to ignore this suggestion if off-base.

Tables: Formatting of column headers.

I see the cost effectiveness, largely due to a reduced vaccine cost and low treatment cost. However, as a clinician clarifying if it comes at a cost of potentially less cases averted and a cost shift to treatment which is cheaper would be nice to clarify.
