# Supplementary material for: Cost-effectiveness analysis of alternative infant and neonatal rotavirus vaccination schedules in Malawi
Source: PLOS Glob Public Health. 2025 Apr 10;5(4):e0004341. doi: 10.1371/journal.pgph.0004341 (PMC11984971; doi:10.1371/journal.pgph.0004341)
Supplement: Attachment — Submitted filename: Response to Reviewers.docx [file pgph.0004341.s017.docx]

**February 6th, 2025**

To the Editor,

Thank you for the opportunity to revise our manuscript, “*Cost-effectiveness Analysis of Alternative Infant and Neonatal Rotavirus Vaccination Schedules in Malawi*”, in response to the reviewers’ feedback. We found the comments to be very helpful in ensuring the clarity of our analysis. Please find the reviewers’ and editor’s comments below in normal text and our responses in bold with changes to the text in red.

We appreciate your time and consideration of this study for publication in *PLOS Global Public Health*.

Sincerely,

**Catherine Wenger, MPH**

(on behalf of the co-authors)

Department of Epidemiology of Microbial Diseases
Yale School of Public Health

catherine.wenger@yale.edu

**Response to reviewers:**

*Reviewer 1*

I would like to congratulate authors for bringing this important evidence for rota virus vaccination policy and program. I have couple of comments.

**Response: We thank the reviewer for their positive assessment and address their specific comments below.**

Point 1 - I would be interested know about the possibility of such analysis for other WHO pre-qualified Rota vaccines which are cheaper that Rotarix (Rotavac & Rotasiil), in fact Rotavac has neonatal strain.

**Response: We chose not to include Rotavac and Rotasiil in this analysis because we do not have sufficient data on the potential efficacy/effectiveness of these vaccines in Malawi or data that directly compares Rotarix to these other vaccines in a clinical trial setting. We also do not have the data yet on the neonatal formulation of the Rotavac vaccine, whereas we use published data on a phase II trial of the neonatal RV3-BB vaccine conducted in Malawi. We describe this limitation in the Discussion in Lines 693-700 of the tracked changes manuscript:**

**“Three other vaccines on the market are administered in three-dose schedules, two of which are cheaper per dose than Rotarix(17, 37). We did not consider these less expensive three-dose vaccines for comparison to the Rotarix schedules because there is no robust evidence on how the vaccine effectiveness of each of these three-dose vaccines compares to two-dose Rotarix. Assuming the other rotavirus vaccines’ effectiveness was comparable to the modeled impact of the three-dose Rotarix schedule, we may find other vaccines on the market are more cost-effective than the current two-dose schedule simply because they are cheaper.”**

**This study also provides a framework for comparing the potential impact and cost-effectiveness of different rotavirus vaccines and/or delivery strategies in other countries. We are in the process of extending this analysis across LMICs more broadly, which requires making assumptions about the efficacy of these vaccines in countries without data, but that is beyond the scope of this current analysis, which focuses on the data available for Malawi.**

Point 2 - Why the booster dose simulation showed aversion of only 400 deaths. Although I am not an expert of such modelling, by logic booster dose should add more effect due to better immune response and due to age accumulated effects.

**Response: The booster dose is administered at 40 weeks and provides immunity for older children who are less likely to die from rotavirus infection. The booster strategy averts more non-severe cases because the age group being protected is more likely to experience non-severe disease than severe disease. In this model, we assume non-severe disease does not result in death (only moderate-to-severe disease does). Therefore, while the booster strategy averts more cases overall, it is not averting as many deaths as other strategies. We added additional explanations to clarify this [Lines 659-664 in tracked changes manuscript]:**

**“While the 6/10/40 strategy averted more cases overall, and the difference in overall cost is marginal, the booster dose primarily averts non-severe cases which are assumed to be non-fatal. The 6/10/14 strategy averted more moderate-to-severe cases by protecting younger children who are more likely to develop severe disease. Therefore, the 6/10/14 strategy averted more DALYs and deaths.”**

**Furthermore, we acknowledge some limitations to the assumptions we make. In particular, we assume the same response rate to a third dose of Rotarix whether it is administered at 14 weeks or 40 weeks (due to the lack of data for the latter). This is noted on Lines 664-668:**

**“However, we assumed the probability of responding to the third dose is the same at 14 and 40 weeks. Children who failed to respond to the first two doses may be more likely to respond to a third dose administered later due to less interference from maternal antibodies, co-administered OPV, better nutritional status, or other factors impacting the immune response(34, 35).”**

*Reviewer 2*

Thank you for allowing me to review this work. I enjoyed the opportunity to learn a lot from the modeling the team performed. While I am not trained in modeling to this level, I did read this from a perspective as a practicing ID specialist with time overseas supporting community epi and economic work to support potential vaccine study. I think the paper is great, and hope that my input is helpful. The below suggestions are largely simple suggestions to consider. Great work!

**Response: We thank the reviewer for their positive comments and address their suggestions below.**

Point 1 - Line 28-This aim statement is a little confusing, but becomes clear in the introduction, consider using a version of the background primary aim statement.

**Response: We agree with the reviewer and adjusted this aim to better match the aim described in the Introduction, as pointed out in Point 3.**

**[Lines 29-33]**

**“This study aimed to identify the optimal rotavirus vaccination strategy for Malawi by modeling vaccine impact and cost-effectiveness comparing the current two-dose Rotarix vaccine schedule to two alternative vaccine delivery schedules and a next-generation neonatal vaccine (RV3-BB) from 2025-2034.”**

Point 2 - Line 39- Attempt better parallel description of the findings. I’m a little confused by the results and the way they are summarized leading up to the final estimated cost/DALY for the neonatal vaccine. Would it be permissible to match the cost/DALY for the respective current Rotarix regimen and the studied phase II regimen, and then transition to a statement comparing cost differences in savings to achieve similar benefit on the population/societal level? It reads as though the new vaccine would result in a lower benefit than keeping with and adding in a booster dose.

**Response: We agree with the reviewer and have revised the abstract to improve the parallel description of the findings [Lines 38-119]:**

**“Over a ten-year time horizon, the current two-dose Rotarix strategy is predicted to avert over 1.5 million cases and 90,000 disability-adjusted life-years (DALYs) compared to no vaccination and is cost-effective at willingness-to-pay (WTP) thresholds above $105 per DALY averted from the government perspective. Adding a third dose at 14 weeks could avert an additional 1 million cases and 38,000 DALYs, while switching to the neonatal RV3-BB vaccine could avert 1.1 million cases and 41,000 DALYs compared to the current strategy. Whereas adding a third dose of Rotarix would cost $4.1-4.9 million, switching to the neonatal vaccine is expected to save $3.7 million compared to the current strategy. Considering the neonatal vaccine is not yet available, adding a third dose of Rotarix at 14 weeks of age is cost-effective at WTP thresholds above $138 per DALY averted. The neonatal vaccine offers a more cost-effective alternative to Malawi’s current rotavirus vaccine, while adding a third dose to the current strategy also provides substantial benefits.”**

Point 3 - Line 83- References a slightly different primary aim than that mentioned in the abstract, consider replacing line 28 with this aim statement inserting the Rotarix 2 dosing strategy comparison

**Response: We agree with the reviewer and adjusted the aim in the abstract to better match this aim in connection to Point 1.**

Point 4 - Line 86- Almost reads like it would be better fit in the methods section, discussion of how you down selected regimens to study at this point seems premature. I think it may read better if the reasons you chose this aim are listed without mention of this process in this section.

**Response: We agree with the reviewer that some of the information in this paragraph is better located in the Methods section. However, we do think that it is important and appropriate to mention the policy questions that our analysis seeks to address in the last paragraph of the Introduction. We therefore left the policy questions this study addresses in the Introduction and moved more technical information to the Methods section.**

**[Moved to Lines 266-267 in the Methods]**

**“We then determined each strategy's cost from the government and societal perspectives and its health outcomes in averting DALYs.”**

Point 5 - Line 239 consider qualifying this 6/10/40 statement with “additional” or similar adjectives to help

**Response: We agree with the reviewer and added qualifiers to clarify this statement comparing the 6/10/14 and 6/10/40 strategies.**

**[Lines 377-378]**

**“… while the 6/10/40 schedule also averted an additional 1 million cases but only 800 deaths (Table S3).”**

Point 6 - Line 242- Are these 1.1 million additional cases averted? Apologies, I’m a clinician and trying to move this quickly, qualifying the values as additional or less, helps me put it into context of the concerns raised about a plateauing of impact. The way it reads now, I may be trading averted cases for cost savings. Visually in the violin plots it looks like it maybe outperforms the other 3 dose regimens.

**Response: Yes, these are additional cases averted by the neonatal strategy compared to the current two-dose strategy. In the case of the neonatal strategy, we are gaining health benefits in addition to saving money compared to the current strategy. We added text to clarify that these are additional health benefits gained by using the neonatal strategy.**

**[Lines 381-382]**

**“…and 1.1 million additional cases, 40,000 additional DALYs, and 1,200 additional deaths compared to the current two-dose Rotarix strategy.”**

Point 7 - Methods- It may be helpful to define threshold for “dominate” strategy like establishing thresholds for statistical significance. Again, this comes from the standpoint of a clinically active ID specialist, and this may be common vernacular for conformity modeling. Feel free to ignore this suggestion if off-base.

**Response: Thank you for this comment. By convention in cost-effectiveness analysis, we label a strategy as “dominated” by another strategy when it both costs more and provides less health benefit. Decision-makers aiming to maximize benefits would not choose to allocate resources to a dominated strategy. Since dominance depends on the relative costs and benefits of the strategies being compared, we cannot assign a static threshold like those used for statistical significance. We have added a definition and reference to clarify the meaning of dominated strategies.**

**[Lines 416-420]**

**“By convention, we label a strategy as “dominated” by one or more other strategies when it is more expensive and averts fewer DALYs than the comparative “dominant” strategies(30).** **In this case, the Rotarix strategies all cost more and avert fewer DALYs than the neonatal strategy and therefore should not be chosen over the neonatal strategy (Table 3).”**

**In addition, we expanded the methods section to describe incremental net benefit (ΔNB) calculations, which, like incremental cost-effectiveness ratios (ICERs), indicate which strategies are dominated using the difference in costs and health benefits between two strategies. ΔNB evaluates the net benefit of a strategy across all willingness-to-pay (WTP) thresholds. In Figure 3, one strategy will become more cost-effective than the previously plotted strategy when the WTP exceeds the ICER of the previously dominant strategy. The Y axis represents the probability of each strategy having the highest net benefit, which serves as a measure of certainty about its cost-effectiveness at a given WTP. We added the equation used to calculate the ΔNB of each vaccine strategy in the Methods section to provide context for the graphs in Figure 3.**

**[Lines 357-361]**

**“The incremental net benefit (ΔNB) of each alternative vaccination strategy compared to the current strategy is calculated as:**

**ΔNB = ΔE*WTP - ΔC,**

**where ΔE is the DALYs averted by a vaccination strategy, and ΔC is the incremental costs of the strategy compared to the status quo.”**

**We also highlighted the probability that each strategy has the highest net benefit to further emphasize this measure of certainty and explain it in relation to the WTP threshold in Figure 3.**

**[Lines 424-427]**

**“At a WTP of 0.5x Malawi’s GDP per capita, the neonatal strategy had an 83% probability of yielding the highest net benefit from the government perspective and an 82% probability from the societal perspective (Fig 3a,b, Fig S4a,b).”**

**[Lines 509-511]**

**“At a WTP of 0.5x Malawi’s GDP per capita, the Rotarix 6/10/14 strategy had a 93% probability of yielding the highest net benefit from the government and societal perspectives (Fig 3c,d, Fig S4c,d).”**

Point 8 - Tables: Formatting of column headers.

**Response: The column headers in Tables 2, 3, and 4 were re-formatted to be consistent within and across tables. All headers now wrap to two lines.**

Point 9 - I see the cost effectiveness, largely due to a reduced vaccine cost and low treatment cost. However, as a clinician clarifying if it comes at a cost of potentially less cases averted and a cost shift to treatment which is cheaper would be nice to clarify.

**Response: We agree with the reviewer that it would be good to clarify the drivers for finding the neonatal vaccine cost-effective. A clarifying sentence has been added to the Discussion.**

**[Lines 607-609]**

**“This finding is driven by the neonatal vaccine's predicted lower cost per dose and the reduced treatment costs resulting from averting more moderate-to-severe cases than any other strategy.”**

**Response to the Editor:**

Point 1 - Please send a completed 'Competing Interests' statement, including any COIs declared by your co-authors. If you have no competing interests to declare, please state "The authors have declared that no competing interests exist". Otherwise please declare all competing interests beginning with the statement "I have read the journal's policy and the authors of this manuscript have the following competing interests:"

**Response: We have checked the Competing Interests statement for accuracy and have updated it with the required language in the submission.**

Point 2 - Thank you for uploading your study's underlying data set. Unfortunately, the repository you have noted in your Data Availability statement does not qualify as an acceptable data repository according to PLOS's standards. At this time, please upload the minimal data set necessary to replicate your study's findings to a stable, public repository (such as figshare or Dryad) and provide us with the relevant URLs, DOIs, or accession numbers that may be used to access these data.

**Response: The dataset and code accompanying this manuscript were published on Dryad and can be found at the following link:** [**https://doi.org/10.5061/dryad.1jwstqk5b**](https://doi.org/10.5061/dryad.1jwstqk5b)

**This has been updated in the submission and manuscript (Line 737-740)**

Point 3 - Please provide separate figure files in .tif or .eps format.

**Response: All figures have been converted to .tif format and processed through PACE diagnostics.**

Point 4 - Please provide an Author Summary. This should appear in your manuscript between the Abstract (if applicable) and the Introduction, and should be 150–200 words long.

**Response: An author summary has been included in the manuscript file between the Abstract and Introduction sections.**

**[Lines 121-135]**

**Author Summary: Rotavirus is a significant cause of severe diarrhea and death due to diarrhea in young children worldwide, particularly in low- and middle-income countries. Despite high vaccine coverage, current live oral rotavirus vaccines are less effective in low-income settings such as Malawi. We explored several vaccine strategies that may improve the impact of routine rotavirus vaccination on disease burden in Malawi. Our epidemiological model simulated the current two-dose Rotarix vaccine strategy and compared to no vaccination, a three-dose neonatal vaccine in development, and adding a third dose of Rotarix at different ages. We then utilized a cost-effectiveness model to predict the costs and disability-adjusted life years incurred by each strategy to determine the optimal strategy. Our results indicate that the current rotavirus vaccination program in Malawi is cost-effective, but adding a third dose at 14 weeks could provide additional benefits. The neonatal vaccine, once available, is likely to be the most cost-effective option and is cost-saving relative to the current strategy. These findings aim to guide policymakers in Malawi and offer a framework for similar analyses in other countries.**

Point 5- Please ensure that all Figure files have corresponding citations and legends within the manuscript. Currently, Figures 4 and 5 in your submission file inventory does not have an in-text citation. If the figure is no longer to be included as part of the submission, please remove it from the file inventory.

**Response: Figure 3 (was Figure 4) has in-text citations on lines 427, 508, 531, 533, 549, and 555. The caption is on lines 440-450. Figure 5 has in-text citations on lines 576 and 578. The caption is on lines 581-588.**

Point 6 - Please review your reference list to ensure that it is complete and correct. If you have cited papers that have been retracted, please include the rationale for doing so in the manuscript text, or remove these references and replace them with relevant current references. Any changes to the reference list should be mentioned in the rebuttal letter that accompanies your revised manuscript. If you need to cite a retracted article, indicate the article’s retracted status in the References list and also include a citation and full reference for the retraction notice.

**Response: The references have been reviewed, and none of them have been retracted. The citation manager we used did not format the page numbers of some of the references correctly, so this has been corrected. The tracked changes are not shown in the manuscript, presumably because the changes were made in the citation manager and auto-populated in the document.**
